# Supplementary material for: A Systematic Review of the Use of Intraoral Scanning for Human Identification Based on Palatal Morphology
Source: Diagnostics (Basel). 2024 Mar 1;14(5):531. doi: 10.3390/diagnostics14050531 (PMC10930713; doi:10.3390/diagnostics14050531)
Supplement: Supplementary file 1 [file diagnostics-14-00531-s001.zip › Supplement S2-Search Strategy.pdf]

Supplementary File S2:Search Strategy

Search Date – 12/12/2023

PubMed - ("Forensic Sciences"[Mesh] OR Forensic* OR "Human identification" OR "human verification") AND (digital OR 3D OR "Intraoral scanner" OR "Imaging, Three-Dimensional"[Mesh]) AND ("Palate, Hard"[Mesh] OR Palatal* OR Palate) AND (Twins[Mesh] OR twins OR sibling* OR Siblings[Mesh] OR family OR Family[Mesh] OR heredity OR hereditary OR Heredity[Mesh])

Embase - ('Forensic Sciences'/exp OR Forensic* OR 'Human identification' OR 'human verification') AND (digital OR 3D OR 'Intraoral scanner' OR 'Imaging, Three-Dimensional'/exp) AND ('Palate, Hard'/exp OR Palatal* OR Palate) AND (Twins/exp OR twins OR sibling* OR Siblings/exp OR family OR Family/exp OR heredity OR hereditary OR Heredity/exp)

Web of Science - ("Forensic Sciences" OR Forensic* OR "Human identification" OR "human verification") AND (digital OR 3D OR "Intraoral scanner" OR "Imaging, Three-Dimensional") AND ("Palate, Hard" OR Palatal* OR Palate) AND (Twins OR twins OR sibling* OR Siblings OR family OR Family OR heredity OR hereditary OR Heredity)

Dentistry and Oral Sciences - ((MH "Forensic Sciences+") OR Forensic* OR "Human identification" OR "human verification") AND (digital OR 3D OR "Intraoral scanner" OR (MH "Imaging, Three-Dimensional+")) AND ((MH "Palate, Hard+") OR Palatal* OR Palate) AND ((MH Twins+) OR twins OR sibling* OR (MH Siblings+) OR family OR (MH Family+) OR heredity OR hereditary OR (MH Heredity+))

Google Scholar – Intraoral scans of the palate for human identification
