# Supplementary material for: A Systematic Review of the Use of Intraoral Scanning for Human Identification Based on Palatal Morphology
Source: Diagnostics (Basel). 2024 Mar 1;14(5):531. doi: 10.3390/diagnostics14050531 (PMC10930713; doi:10.3390/diagnostics14050531)
Supplement: Supplementary file 1 [file diagnostics-14-00531-s001.zip › Supplement S3-Quality Assessment.pdf]

Supplementary File S3: Critical Appraisal Skills Programme (CASP) – cohort study checklist

	Taneva et al	Simon et al (2021)	Simon et al (2022)	Simon et al (2020)	Bjelopavlovic et al	Mikolicz et al	Simon et al (2023)
1. Did the study address a clearly focused issue?	Yes	Yes	Yes	Yes	Yes	Yes	Yes
2. Was the cohort recruited in an acceptable way?	Yes	Yes	Yes	Yes	Yes	Yes	Yes
3. Was the exposure accurately measured to minimize bias?	Yes	Yes	Yes	Yes	Yes	Yes	Yes
4. Was the outcome accurately measured to minimize bias?	Yes	Yes	Yes	Yes	Yes	Yes	Yes
5. (a) Have the authors identified all important confounding factors?	No	No	No	No	Can't tell	Yes	Yes
5. (b) Have they taken account of the confounding factors in the design and/or analysis?	Yes	No	No	No	No	Yes	No
6. (a) Was the follow up of subjects complete enough?	Yes	NA	NA	NA	Yes	Yes	NA

6. (b) Was the follow up of subjects long enough?	Can't tell	NA	NA	NA	Can't tell	Can't tell	NA
7. What are the results of this study?	3D palatal landmarks could be used for human identification as they are not influenced by time as well as orthodontic treatment	The 3D digital palatal model could serve as a reliable tool for human identification	3D data containing only palatal height, width, and depth without surface morphology could assist with human identification	Monozygotic twin siblings are highly distinguishable from one another and may represent individuality for the entire human community	Palatal fold pairs do not differ at different time points and are highly individual-specific	The anterior area of the palate is a good candidate for identification owing to its inclusion in antemortem scans and good reproducibility	3D deviations between replicate scans of the same individual were much lesser than between-sibling values.
8. How precise are the results?	Yes	Yes	Yes	Yes	Yes	Yes	Yes
9. Do you believe the results?	Yes	Yes	Yes	Yes	Yes	Yes	Yes
10. Can the results be applied to the local population?	Yes	Yes	Yes	Yes	Yes	Yes	Yes
11. Do the results of this study fit with other available evidence?	Yes	Yes	Yes	Yes	Yes	Yes	Yes
12. What are the implications of this study for practice?	Yes	Yes	Yes	Yes	Yes	Yes	Yes
